# Targeted disruption of dual leucine zipper kinase and leucine zipper kinase promotes neuronal survival in a model of diffuse traumatic brain injury

**DOI:** 10.1186/s13024-019-0345-1

**Published:** 2019-11-27

**Authors:** Derek S. Welsbie, Nikolaos K. Ziogas, Leyan Xu, Byung-Jin Kim, Yusong Ge, Amit K. Patel, Jiwon Ryu, Mohamed Lehar, Athanasios S. Alexandris, Nicholas Stewart, Donald J. Zack, Vassilis E. Koliatsos

**Affiliations:** 1 Department of Ophthalmology, University of California, San Diego, La Jolla, 92037 USA; 2 Department of Neurology, The Johns Hopkins University School of Medicine, Baltimore, MD 21205 USA; 3 Division of Neuropathology, Department of Pathology, The Johns Hopkins University School of Medicine, Baltimore, MD 21205 USA; 4 Department of Ophthalmology, The Johns Hopkins University School of Medicine, Baltimore, MD 21205 USA; 5 Department of Otolaryngology-Head and Neck Surgery, The Johns Hopkins University School of Medicine, Baltimore, MD 21205 USA; 6 Department of Molecular Biology and Genetics, The Johns Hopkins University School of Medicine, Baltimore, MD 21205 USA; 7 The Solomon H. Snyder Department of Neuroscienc, The Johns Hopkins University School of Medicine, Baltimore, MD 21205 USA; 8 Institute of Genetic Medicine, The Johns Hopkins University School of Medicine, Baltimore, MD 21205 USA; 9 Department of Psychiatry and Behavioral Sciences, The Johns Hopkins University School of Medicine, Baltimore, MD 21205 USA

**Keywords:** Traumatic axonal injury, Concussion, Cell death, Traumatic brain injury, Optic neuropathy, Dual leucine zipper kinase, DLK, LZK, Retinal ganglion cell

## Abstract

**Background:**

Traumatic brain injury (TBI) is a major cause of CNS neurodegeneration and has no disease-altering therapies. It is commonly associated with a specific type of biomechanical disruption of the axon called traumatic axonal injury (TAI), which often leads to axonal and sometimes perikaryal degeneration of CNS neurons. We have previously used genome-scale, arrayed RNA interference-based screens in primary mouse retinal ganglion cells (RGCs) to identify a pair of related kinases, dual leucine zipper kinase (DLK) and leucine zipper kinase (LZK) that are key mediators of cell death in response to simple axotomy. Moreover, we showed that DLK and LZK are the major upstream triggers for JUN N-terminal kinase (JNK) signaling following total axonal transection. However, the degree to which DLK/LZK are involved in TAI/TBI is unknown.

**Methods:**

Here we used the impact acceleration (IA) model of diffuse TBI, which produces TAI in the visual system, and complementary genetic and pharmacologic approaches to disrupt DLK and LZK, and explored whether DLK and LZK play a role in RGC perikaryal and axonal degeneration in response to TAI.

**Results:**

Our findings show that the IA model activates DLK/JNK/JUN signaling but, in contrast to axotomy, many RGCs are able to recover from the injury and terminate the activation of the pathway. Moreover, while DLK disruption is sufficient to suppress JUN phosphorylation, combined DLK and LZK inhibition is required to prevent RGC cell death. Finally, we show that the FDA-approved protein kinase inhibitor, sunitinib, which has activity against DLK and LZK, is able to produce similar increases in RGC survival.

**Conclusion:**

The mitogen-activated kinase kinase kinases (MAP3Ks), DLK and LZK, participate in cell death signaling of CNS neurons in response to TBI. Moreover, sustained pharmacologic inhibition of DLK is neuroprotective, an effect creating an opportunity to potentially translate these findings to patients with TBI.

## Background

Traumatic brain injury (TBI) affects over 2.5 million people each year in the U.S. alone, and is associated with lasting deficits in motor, sensory, and higher CNS functions [1]. As of yet, there are no therapies that prevent or alter the course of these conditions and, thus, there is an urgent need to better understand the molecular pathways that are responsible for the underlying neuropathology and then develop disease-modifying drugs. A very common pathology in TBI of various causes and degrees of severity is diffuse or traumatic axonal injury (TAI) [2–6]. TAI is thought to be caused by rotational acceleration of the head resulting in extremely rapid axonal stretching in multiple white matter foci in the forebrain and brain stem. We and others have shown that TAI can be modeled in the rodent brain with specifically arranged impact forces [7–12].

A consistent finding in multiple TBI models, including blunt force models such as fluid percussion and impact acceleration (IA) as well as blast, is the presence of TAI in the optic nerve and optic tract [11, 13–15]. This interesting observation, combined with the accessibility and compartmentalized anatomy of the visual system, suggests that retinal ganglion cells (RGCs) are an excellent neuronal system in which to model TAI and explore the underlying cellular and molecular mechanisms.

We have previously identified the key mediators involved in RGC axon injury signaling in primary mouse RGCs with a high-throughput, functional genomic screen. Using small interfering RNAs (siRNAs) to knockdown gene expression, we screened through the mouse genome and, along with others, identified a multi-tiered kinase cascade that is responsible for axon injury signaling in vitro and in vivo [16–19]. This cascade includes the mitogen-activated protein kinase kinase kinases (MAP3Ks) dual leucine zipper kinase (DLK/MAP3K12) and leucine zipper kinase (LZK/MAP3K13), the mitogen-activated protein kinase kinases (MAP2Ks) MKK4/MAP2K4 and MKK7/MAP2K7, and the mitogen-activated protein kinases (MAPKs) JUN N-terminal kinase (JNK) 1–3. In response to optic nerve crush (ONC), DLK is robustly upregulated at the site of axonal injury and is transported to the soma where it initiates a toxic genetic program mediated by multiple transcription factors including JUN [16, 17, 20, 21]. While DLK inhibition is sufficient to reduce cell death after axonal injury, combined DLK and LZK inhibition leads to a more robust protective phenotype suggesting some degree of redundancy [18]. Moreover, there is still uncertainty as to the degree of participation of DLK in the degeneration of axons following injury [16, 17, 22–24]. To the extent that TAI in various models of TBI, including IA, engages this same injury signaling cascade, DLK/LZK inhibition could become part of a neuroprotective treatment strategy for TBI.

In the present study, we explore the activation of DLK/LZK signaling in RGCs in the course of TAI in the mouse visual system inflicted with IA. We then establish the role of this kinase pathway in RGC somal and axonal degeneration with genetic and pharmacological strategies designed to block DLK and LZK. Our results indicate that the DLK/LZK-JNK axis is robustly involved in RGC death associated with TAI in the visual system and suggest a broader role of this kinase cascade in primary axonopathies associated with TBI.

## Methods

### Experimental subjects and IA procedures

Eight-week-old male C57BL/6 J wild-type mice and transgenic *Dlk*^*fl/fl*^ and *Dlk*^*fl/fl*^*Lzk*^*fl/fl*^ mice were subjected to IA or sham injury. Male mice were chosen such as to avoid the confounding effects of sex hormones on injury outcomes [25–29]. Wild-type mice and founders were purchased from Charles River Laboratories (Wilmington, MA). Animals were housed in a vivarium with a 12-h light/12-h dark cycle and given ad libitum access to food and water. All animal handling as well as surgical and postoperative procedures were carried out according to protocols approved by the Animal Care and Use Committee of the Johns Hopkins Medical Institutions.

Impact acceleration injury was performed with height-weight settings generating kinetic energy of 0.45–0.5 J upon impact, essentially as described [11, 12] **(**Table 1**)**. Immediately prior to injury, the cranium was exposed and a 5 mm-thick stainless-steel disc was glued onto the skull midway between bregma and lambda sutures. Surgical procedures and injury were performed under aseptic conditions with gas anesthesia (isoflurane: oxygen: nitrous oxide = 1:33:66). Immediately after injury the disc was removed the skull was checked under the surgical microscope for skull fractures. The rare animals with skull fractures were excluded from further study because such events introduce injuries variables that cannot be easily controlled. Sham animals were subjected to the same procedures, but without the weight drop. The scalp incision was closed with surgical staples, and the animal was returned to cage.

**Table 1:** Impact acceleration condition and survival time for each experimental group

Experiments/groups	Experimental history, survival times post-IA	Procedures
1.Characterization of initial injury in the optic nerve - general	Sham (*n* = 3), 4 h post-IA (*n* = 3), 24 h post-IA (*n* = 3) IA:40 g × 1 m or 60 g × 0.85 m	IHC for APP (TAI) and IgG (BBB disruption)
2. Characterization of initial injury in the optic nerve - CLARITY	CTB injection immediately post-IA, survival 2 d post-IA (*n* = 3) IA: 40 g × 1 m -– 60 g × 0.85 m	CLARITY
3. Characterization of traumatic axonopathy - general neuropathology in the optic nerve and tract	7 d post-IA (*n* = 3) IA:40 g × 1 m or 60 g × 0.85 m	Gallyas silver IHC for neuroinflammation (IBA1)
4. Characterization of traumatic axonopathy - death of RGCs in retina	Sham (*n* = 6), 2 wk. post-IA (*n* = 7), 4 wk. post-IA (*n* = 5) IA:40 g × 1 m	IHC for γ Synuclein Cell counts on retinal wholemounts
5. Characterization of traumatic axonopathy - axonal degeneration in optic nerve	Sham (*n* = 6), 2 wk. post-IA (*n* = 7), 4 wk. post-IA (*n* = 5) IA:40 g × 1 m	Embedding of optic nerve tissues in epoxy resin, semithin sectioning, toluidine blue staining
6. Induction of DLK-JNK pathway in RGCs after injury	24 h (*n* = 3), 3 d post-IA (*n* = 3) IA:40 g × 1 m	IHC for DLK, p-JUN in retinal sections
7. Time course of DLK-JNK induction in RGCs after injury	Sham (*n* = 4), 24 h post-IA (*n* = 10), 3 d post-IA (*n* = 5), 7 d post-IA (*n* = 4), 14 d post-IA (*n* = 3) IA:40 g × 1 m	IHC for γ Synuclein and p-JUN Cell counts on retinal wholemounts
8. Time course of activation of distinct member of DLK-JNK pathway in RGCs after injury	Sham (*n* = 3), 24 h post-IA (*n* = 3), 3 d post-IA (*n* = 3), 7 d post-IA (*n* = 3) IA:40 g × 1 m	Standard Western blotting
9. Interventional studies - genetic deletion of *Dlk* on *Dlk*^*fl/fl*^ mice and effects on DLK-JNK pathway activation and RGC survival	AAV2-Cre-GFP into one eye AAV2-GFP into fellow eye (*n* = 4 each, injections 2 wk. pre-IA, euthanasia 3 days post-IA) IA:60 g × 0.85 m	IHC for p-JUN Cell counts on retinal wholemounts
	AAV2-Cre-GFP into one eye AAV2-GFP into fellow eye (*n* = 7 each, injections 2 wk. pre-IA, euthanasia 4 wk. post-IA) IA:60 g × 0.85 m	IHC for RBPMS Cell counts on retinal wholemounts
10. Interventional studies - genetic deletion of *Dlk* and *Lzk* on *Dlk*^*fl/fl*^*Lzk*^*fl/fl*^ and effects on DLK-JNK pathway activation and RGC survival	AAV2-Cre-GFP into one eye AAV2-GFP into fellow eye (*n* = 4 each, injections 2 wk. pre-IA, euthanasia 3 day post-IA) IA:60 g × 0.85 m	IHC for p-JUN Cell counts on retinal wholemounts
	AAV2-Cre-GFP into one eye AAV2-GFP into fellow eye (*n* = 7 each, injections 2 wk. pre-IA, euthanasia 4 wk. post-IA) IA:60 g × 0.85 m	IHC for RBPMS Cell counts on retinal wholemounts
11. Interventional studies - pharmacologic kinase inhibition with sunitinib and effects on DLK-JNK pathway activation and RGC survival	Drug (*n* = 12) Vehicle (*n* = 14), both groups 3 wk. post-IA IA:40 g × 1 m	IHC for γ Synuclein Cell counts on retinal wholemounts

### Histology, histochemistry, immunohistochemistry and microscopy

At the various survival times listed in Table 1, animals were transcardially perfused with freshly depolymerized, 4% neutral-buffered paraformaldehyde. Eyes were enucleated and fixed for 2 h while optic nerves were immersed in the same fixative overnight at 4 °C. In some cases, brains were also included in the study for the analysis of optic tracts and superior colliculi. Tissues were cryoprotected in 30% sucrose and stored at − 80 °C until further processing. Sagittal brain sections (40 μm), some with attached distal nerves, were prepared in series for Gallyas silver staining for injured/degenerating axons and terminals and immunohistochemistry (IHC) for the microglial marker IBA1. Gallyas silver staining was performed as described [14]. Sagittal retinal sections (10 μm) were prepared on a cryostat and processed in series for immunoperoxidase-based IHC to assay for select members of the DLK-JNK pathway, including DLK, phosphorylated JNK (p-JNK) and phosphorylated JUN (p-JUN). Sagittal sections of the entire optic nerve were used to assay for the presence of axon injury and blood brain barrier (BBB) disruption at early time points post-injury, with amyloid precursor protein (APP) and IgG staining, as described [11]. Retinal flat mounts were prepared as described [11] and used to explore injury responses or survival of RGCs based on p-JUN and γ-synuclein (SNCG) immunofluorescence.

In all experiments involving IHC, sections were first incubated in the primary antibody overnight at 4 °C. For immunoperoxidase staining, after incubation with biotinylated secondary antibody (1:200; Jackson ImmunoResearch, West Grove, PA) and then avidin and biotinylated peroxidase, sections were developed with 3,3′-diaminobenzidine (DAB) (Vectastain Elite ABC Kit; Vector Laboratories Inc., Burlingame, CA). For immunofluorescence, after incubation in secondary antibodies conjugated with Cy3 or Cy2 (1:200; Jackson ImmunoResearch, West Grove, PA) for 2–4 h at room temperature, sections were counterstained with the fluorescent DNA dye 4′, 6-diamidino-2-phenylindole (DAPI) and coverslipped with DPX. Primary antibodies included: anti-IBA1 (1:500; Dako; 019–19,741, Carpinteria, CA or Biocare Medical; CP-290A, Pacheco, CA); DLK (1:200, GeneTex; GTX124127, Irvine, CA); p-JNK (1:200, Cell Signaling Technology; 9251 or 4671, Danvers, MA); p-JUN (1:200, Cell Signaling Technology; 9261 or 3270, Danvers, MA); SNCG (1:600; Abnova; H00006623-M01, Walnut, CA or Genetex; GTX110483, Irvine, CA); RBPMS (1:1000, PhosphoSolutions; 1832-RBPMS or 1830-RBPMS, Aurora, CO); and TUJ1 (1:600, Covance; MMS-435P or PRB-435P, Indianapolis, IN or Abcam; ab18207, Cambridge, MA). Immunostained sections were studied on a Zeiss Axiophot microscope equipped for epifluorescence or a Zeiss LSM 510 inverted confocal microscope. Adobe Photoshop 9.0 software (Adobe Systems, San Jose, CA) was used for image processing.

### CLARITY-based processing of the optic nerve

To explore the location and extent of axonal injury in the optic nerve after IA, the anterograde tracer cholera toxin subunit B (CTB) conjugated with Alexa Fluor® 488 (CTB 488) was injected intravitreally 1 h after IA or sham injury. Optic nerves were then dissected and processed by CLARITY as described [12]. In brief, optic nerves were incubated in a hydrogel containing 2% acrylamide, 0.025% bis-acrylamide, 0.25% VA-044 initiator and 4% PFA in 0.1 M PBS overnight (4 °C). After degassing, samples were incubated in a water bath (37 °C) for 3 h. Polymerized nerves were then transferred to a boric acid solution with 4% SDS and were incubated for 4 days. Prior to imaging, optic nerves were incubated in FocusClear (CellExplorer catalog #FC-101) and mounted for imaging. Transparent optic nerves were imaged with confocal microscopy at 20× on a Zeiss AxioExaminer microscope with a 710NLO module (Carl Zeiss Inc., Oberkochen, Germany) and images were visualized with Imaris software (Bitplane, Concord, MA).

### Semithin-section processing of the optic nerve

Optic nerves from sham- and IA-injured subjects were dissected away from the eyes and brains and treated with a solution containing 4% paraformaldehyde and 0.2% glutaraldehyde for 24 h. After rinsing in 0.1 M phosphate buffer (pH 7.3) for 3–10 min, tissues were immersed in 1% osmium tetroxide for 15 min and stained *en bloc* with 1% uranyl acetate for 1 h. Stained tissues were dehydrated in graded concentrations of ethanol, embedded in Poly/Bed 812 (Polysciences Inc., Warrington, PA) in BEEM® capsules and polymerized at 60 °C for 72 h. Semithin sections (1 μm) were cut transversely from segments of optic nerves caudal to the eyeball and stained with 1% toluidine blue. Myelinated axonal profiles were studied under 100× magnification on a Zeiss Axiophot microscope equipped for epifluorescence (Diagnostic Instruments Inc., Sterling Heights, MI); normal profiles were counted by investigators blinded to experimental history using the optical fractionator probe in the Stereo Investigator® software (Microbrightfield Inc., Williston, VT).

### Immunoblots

To explore the involvement of select members of the DLK-JNK axis in visual TAI after IA, we harvested fresh eyeballs at days 1, 3 and 7 after injury and then dissected the retinas and stored them at − 80 °C. For protein extraction, retinas were sonicated in cell lysis buffer containing 1 mM PMSF (Cell Signaling Technology, Danvers, MA), complete protease inhibitor cocktail and PhosSTOP phosphatase inhibitor cocktail (Roche, Basel, Switzerland) and then incubated for 30 min at room temperature. Solubilized proteins in Laemmli sample buffer were separated on SDS-PAGE gel and then transferred to polyvinylidene fluoride (PVDF) membranes using XCell II blot system (Invitrogen, Carlsbad, CA). Membranes were blocked with 5% Bovine Serum Albumin (BSA) in Tris-buffered saline/0.05% Tween-20 and sequentially incubated in primary antibodies (overnight, 4 °C). In addition to DLK, p-JNK and p-JUN (all at 1:1000) antibodies that were the same as in previous section, we also used antibodies against phosphorylated MKK4 and MKK7 (1:1000, Cell Signaling Technology, Danvers, MA), and a β-actin antibody (1:1000, Cell Signaling Technology, Danvers, MA). HRP-conjugated secondary antibody (1 h at RT) and SuperSignal Chemiluminescent Substrate (Thermo Scientific. Reckford, IL) was used to detect protein signals. Image J (National Institutes of Health) and Prism (GraphPad Software, Inc., La Jolla, CA) were used for quantitation and statistical analysis.

### *Dlk*/*Lzk* knockout in *Dlk*^*fl/fl*^ and *Dlk*^*fl/fl*^*Lzk*^*fl/fl*^ mice

Male *Dlk*^*fl/fl*^ and *Dlk*^*fl/fl*^*Lzk*^*fl/fl*^ mice were subjected to IA as described above [11, 12]. Two weeks prior to injury, subjects were intravitreally injected in one eye with AAV2 expressing Cre-GFP (AAV-Cre) and in the fellow eye with AAV2 expressing GFP (AAV-GFP). In one set of experiments (*n* = 5 per *Dlk*^*fl/fl*^, *Dlk*^*fl/fl*^ *Lzk*^*fl/f*^ and wild-type mice), 3 days after injury, retinal flat mounts were immunostained for p-JUN and taken for counts of immunoreactive neurons as laid out in previous sections, with the AAV2-GFP-injected eye serving as control. In another experimental scenario (*n* = 9–10 per *Dlk*^*fl/fl*^, *Dlk*^*fl/fl*^ *Lzk*^*fl/fl*^ and wild-type mice) animals were allowed to survive for 3 weeks and retinal flat mounts were processed for counts of SNCG-positive or RBPMS-positive RGCs as described in previous Sections.

### DLK-JNK inhibition with the protein kinase inhibitor sunitinib

Sunitinib stock solution was made by dissolving drug powder in dimethylsulfoxide (DMSO) at a concentration of 120 mg/ml and was stored at − 20 °C. At the time of drug administration, stock solution was diluted with saline to a final concentration of 6 mg/ml and injected intraperitoneally into mice (60 mg/kg). Sunitinib was administered 24 and 4 h prior to injury, and then once more immediately after the injury (for the evaluation of p-JUN expression in RGCs) or daily for 3 weeks starting immediately after the injury (for the evaluation of RGC survival). Control animals received injections of vehicle solution.

### RGC counts in retinal flatmounts

The four quadrants of retinal mounts immunolabeled with SNCG and p-JUN were separately imaged with a 20× objective (0.4 numerical aperture) on a Zeiss LSM 510 inverted confocal microscope (Carl Zeiss Inc., Oberkochen, Germany). Images were adjusted for optimal contrast and brightness with Adobe Photoshop 7.0 and Image J was used to count surviving SNCG (+), RBPMS (+) or injured p-JUN (+) RGCs. Cells were counted in 150 × 150 μm fields from three concentric zones of the same width, from the center to the periphery of the retina, using at least 8 fields per each zone. Cell density was calculated by dividing cell numbers by total area surveyed. The ratio of p-JUN (+) RGCs cells was calculated by dividing the number of p-JUN/SNCG double-labeled cells by the total number of SNCG (+) cells. In a number of experiments, i.e. p-JUN response and survival of RGCs in *Dlk*^*fl/fl*^ and *Dlk*^*fl/fl*^*Lzk*^*fl/fl*^ mice and RGC survival in the sunitinib experiment, counts were done using an automated image analysis system (ImageXpress high content imager, Molecular Devices, Sunnyvale CA) using the autofocus and auto-tiling function at 20× magnification followed by automatic quantification with ImageJ.

### Statistical methodologies

Statistical analysis was carried out with one-way ANOVA or *t* test. In the case of ANOVA, significant differences were further analyzed with Tukey’s post hoc test to reveal important main effects or interactions. For counts of axonal number in the characterization of optic neuropathy, numbers were normalized to sham mean. Calculations were performed using Prism 4 (GraphPad Software). Differences were considered significant at *p* < 0.05.

## Results

### Traumatic axonal injury in the visual system is associated with RGC axonopathy and axonal degeneration

**Fig. 1:**
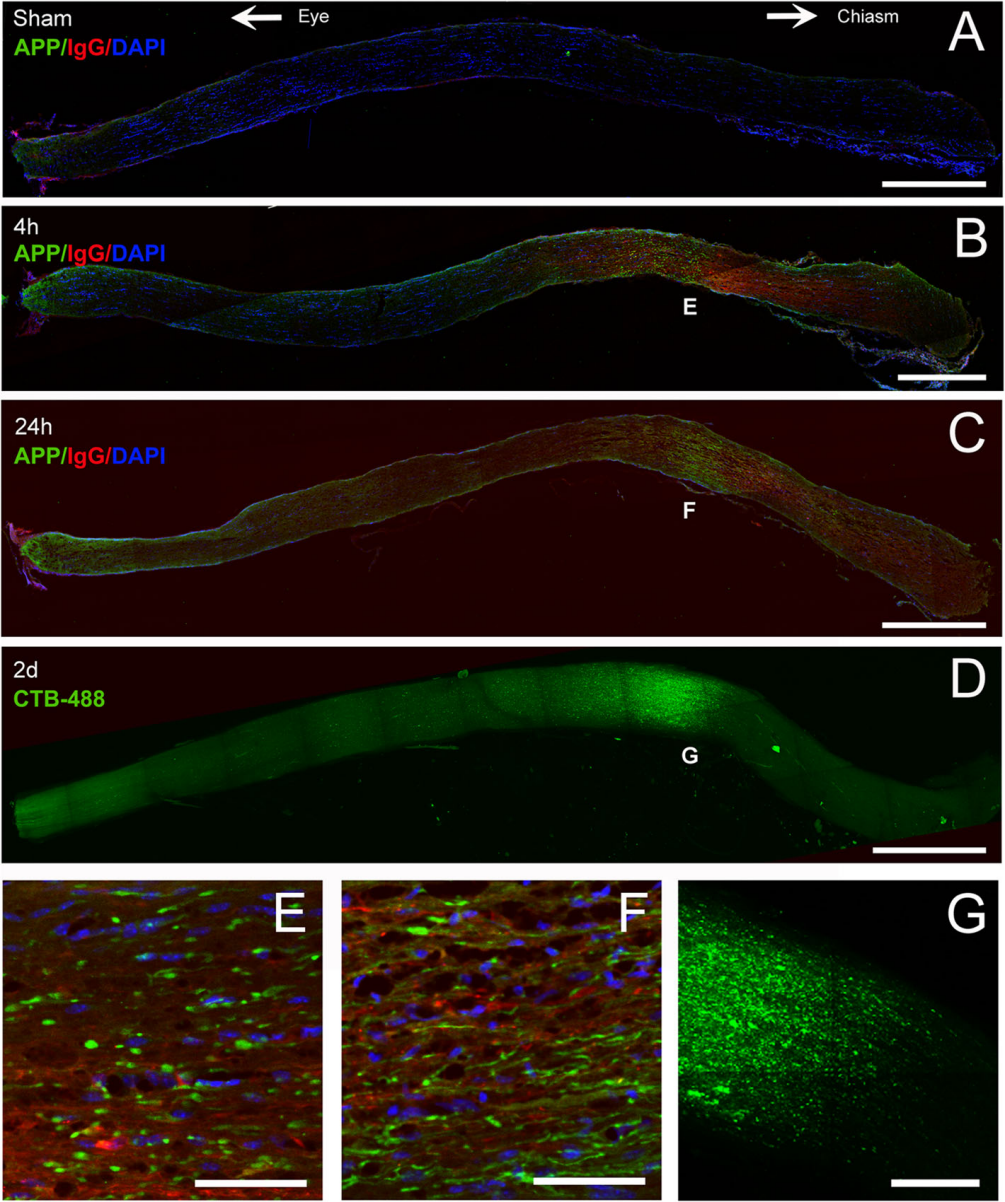
Location of the initial optic nerve disruption injury in IA-injured animals. **a**-**c**. Early axonal and BBB changes as shown with double immunofluorescence for APP (green) and mouse IgG (red). Eyeball is to the left, optic chiasm to the right of panel. Four hours after injury, there are multiple axonal swellings and bulbs (green), evidence of primary TAI at approximately 2/3 of optic nerve from the eyeball. There is also disruption of BBB manifested by IgG leakage (red) in the same area but covering a larger segment of the nerve. There are no APP (+) axonal abnormalities or BBB disruption in the optic nerve of sham mice. Insets show representative lesions in two magnifications at four (**b**) and 24 (**c**) hours post-injury. **d.** Disruption of CTB transport in optic nerve after IA injury: Axonal transport in optic nerve was explored by intravitreally injecting CTB488 1 h after injury. Processed with CLARITY 2 days after injury, CTB-488 (green) transport is interrupted at approximately 2/3 of the optic nerve from the eyeball at exactly the same site as APP (+) axonal abnormalities and BBB alterations in **a**-**c. e**-**f**. Details of axonal abnormalities at the level of initial mechanical nerve disruption. Panels are magnifications of identically labeled regions in **b**, **c**, and **d**. Note classical axonal bulbs 4 h post-injury (**e**), varicosities and undulations at 24 h (**f**), and the interrupted axonal transport of CTB in most axons in (**g**). Scale bars: **a**-**c**, 500 μm; **d**, 550 μm; **e**-**g**, 50 μm

To characterize the initial effect of IA on the optic nerve, sections were immunostained for amyloid precursor protein (APP), a marker of early TAI. As early as 4 h after injury, we found a distinct region of APP-positive swellings and bulbs between the orbital apex and the chiasm (Fig. 1a-c**)**. Amyloid precursor protein colocalized with IgG immunoreactivity, indicating serum extravasation and blood-brain barrier (BBB) disruption (Fig. 1b-c). Axonal abnormalities included classical axon bulbs and varicosities (Fig. 1e-f). When retinas were injected with CTB488 and optic nerves processed with CLARITY to visualize transport in the nerve, we identified CTB488 transport blockade in the same location of the nerve that had APP-positive axonal abnormalities (Fig. 1d, g). These findings indicate that IA produces a focal traumatic injury to RGC axons in the intracranial portion of the optic nerve.

**Fig. 2:**
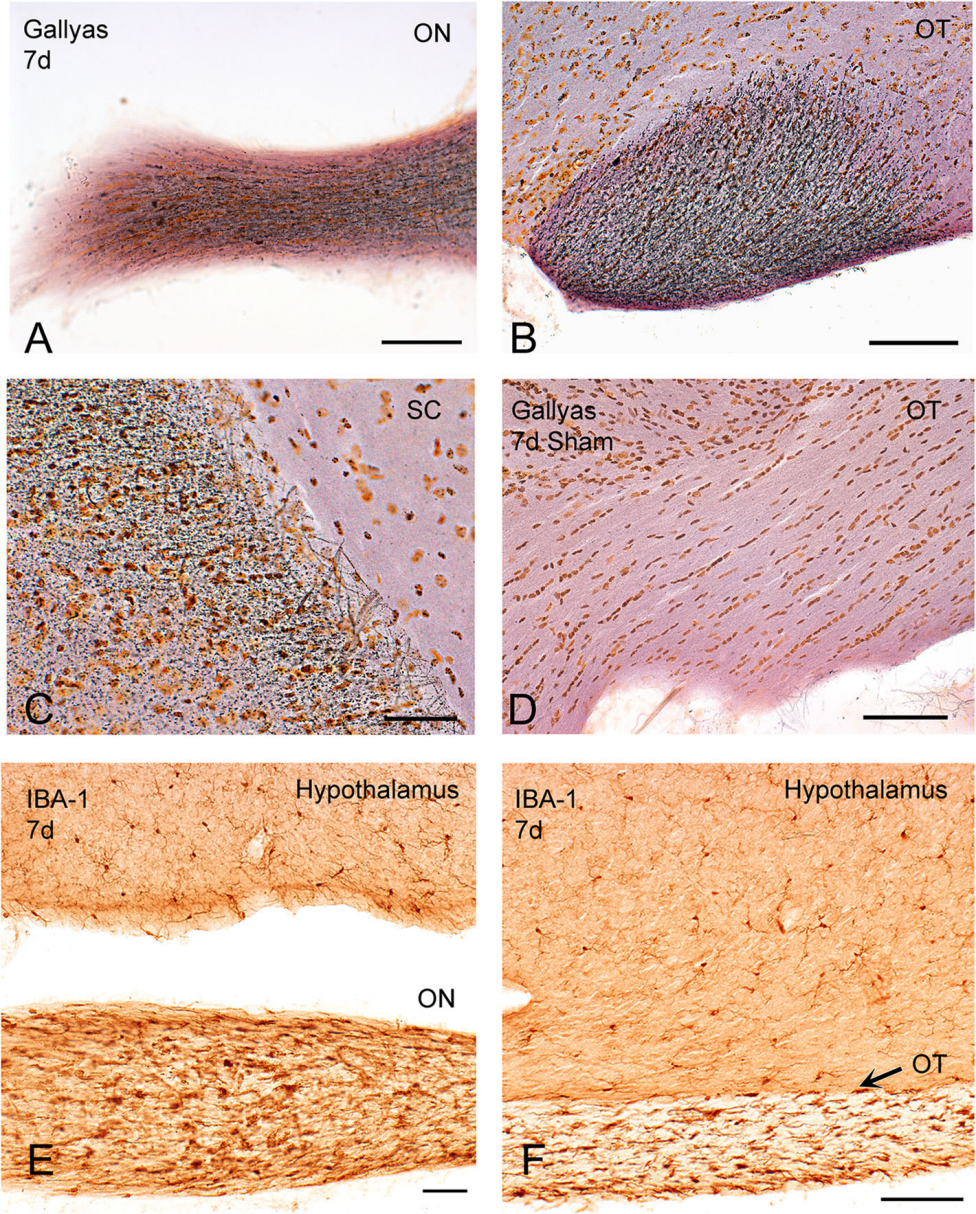
Axonal degeneration (Gallyas silver) and neuroinflammation in the visual system after IA injury. **a**-**d**. Gallyas silver degeneration staining shows axonal pathology (black swellings, lines, trails, dots) 7 days after injury in the optic nerve (ON, **a**), optic tract (OT, **b**), and superior colliculus (SC, **c**). Sham tissues show no silver signal at the same time point (**d**). **e-f.** Neuroinflammatory responses are evident by the presence of hypertrophic IBA1 (+) microglia and microglial nodules in these sections prepared 7 days after injury. Note the striking selectivity of neuroinflammation in the visual system (optic nerve [ON] and optic tract [OT]) by comparing IBA1 (+) profiles in ON and OT to normal resting microglia in overlying hypothalamus. Scale bars: **a-d**, 100 μm; **e**, 80 μm; **f**, 150 μm

**Fig. 3:**
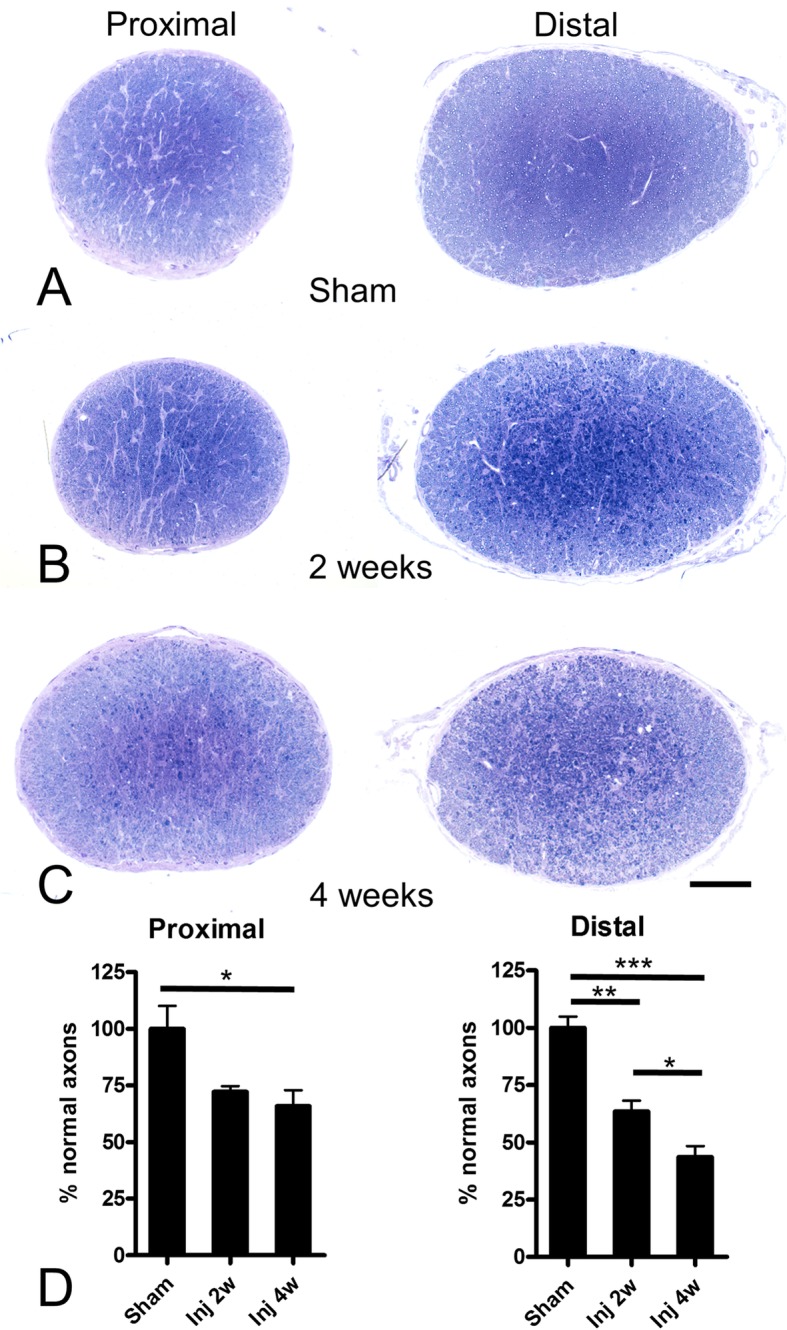
Axonal degeneration in the proximal and distal optic nerve following IA injury. Axonal pathology in the optic nerve of mice was examined with toluidine blue staining of semithin sections from sham (**a**) and 2- or 4-week post-injury cases (**b** and **c**, respectively). The nerve segment proximal to initial traumatic disruption is on the left, distal to disruption segment (close to chiasm) is on the right. Within the first month, there is evident axonopathy, distal more than proximal. In both segments and time points, pathology is more severe at the center of the nerve. Fields are further enlarged for greater cellular detail in Fig. 4. Panel **d** is a bar graph with stereological counts of normal axons in the proximal and distal segments of the optic nerve in the sham condition and at 2 and 4 weeks post injury. Numbers are expressed as percentages of sham because there is a difference in baseline myelinated axons in the proximal and distal segments in this experiment where proximal sections were taken from a plane ~ 0.5–1 mm posterior to the eye. Scale bars: 100 μm

**Fig. 4:**
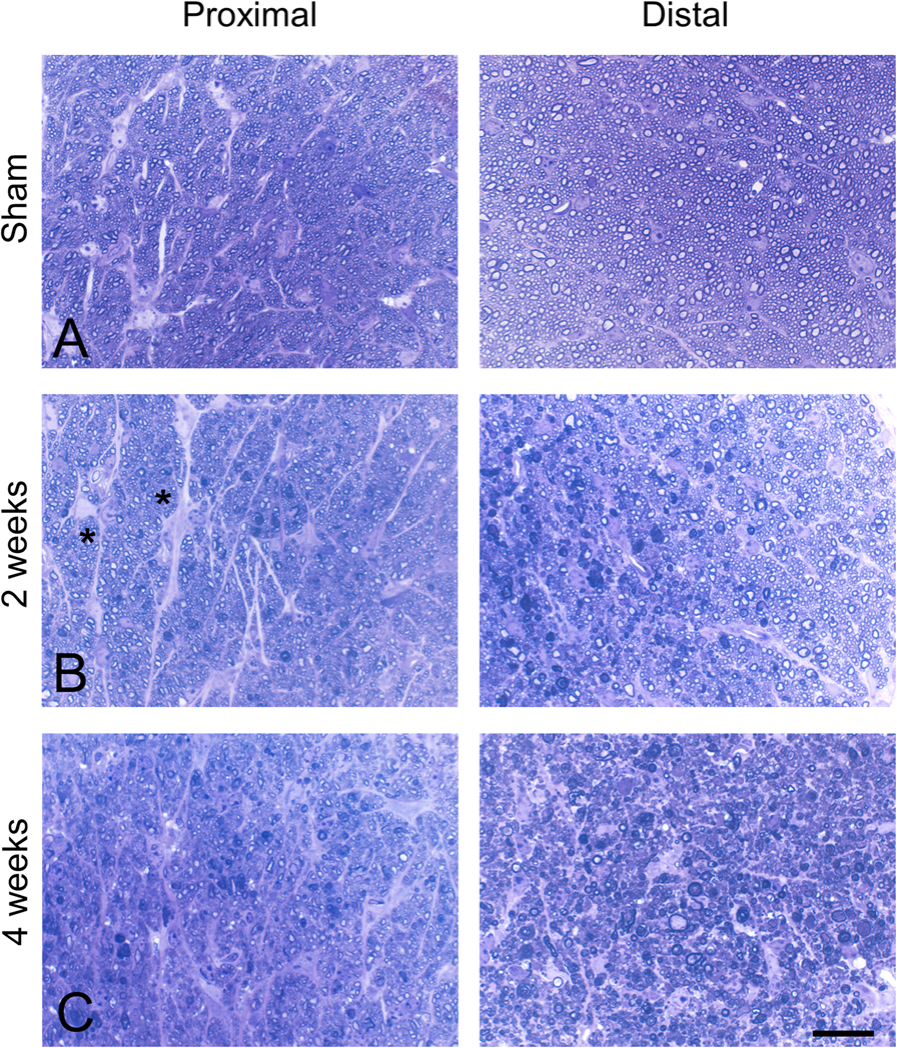
Details of traumatic axonopathy in the optic nerve at the semithin level. Illustrated fields are from the same sections used for Fig. 3. All panels are from toluidine blue-stained semithin sections of the optic nerve proximal to the initial traumatic disruption of the nerve (close to the eye; *left panels*) and distal to traumatic disruption (towards the optic chiasm; *right panels*). The sham condition is illustrated in (**a**), and representative fields from lesioned nerves two and 4 weeks post injury are illustrated in (**b**) and (**c**). The main finding is myelin pathology which is especially severe at 2 weeks distally and 4 weeks proximally. A lot of abnormal myelin signal in the distal segment at 4 weeks is in the form of residual small myelin fragments. Note the prominent presence of astrocytes in the proximal segment that have transformed into hypertrophic, reactive profiles in (**b**) (asterisks). Scale bar: 10 μm

**Fig. 5:**
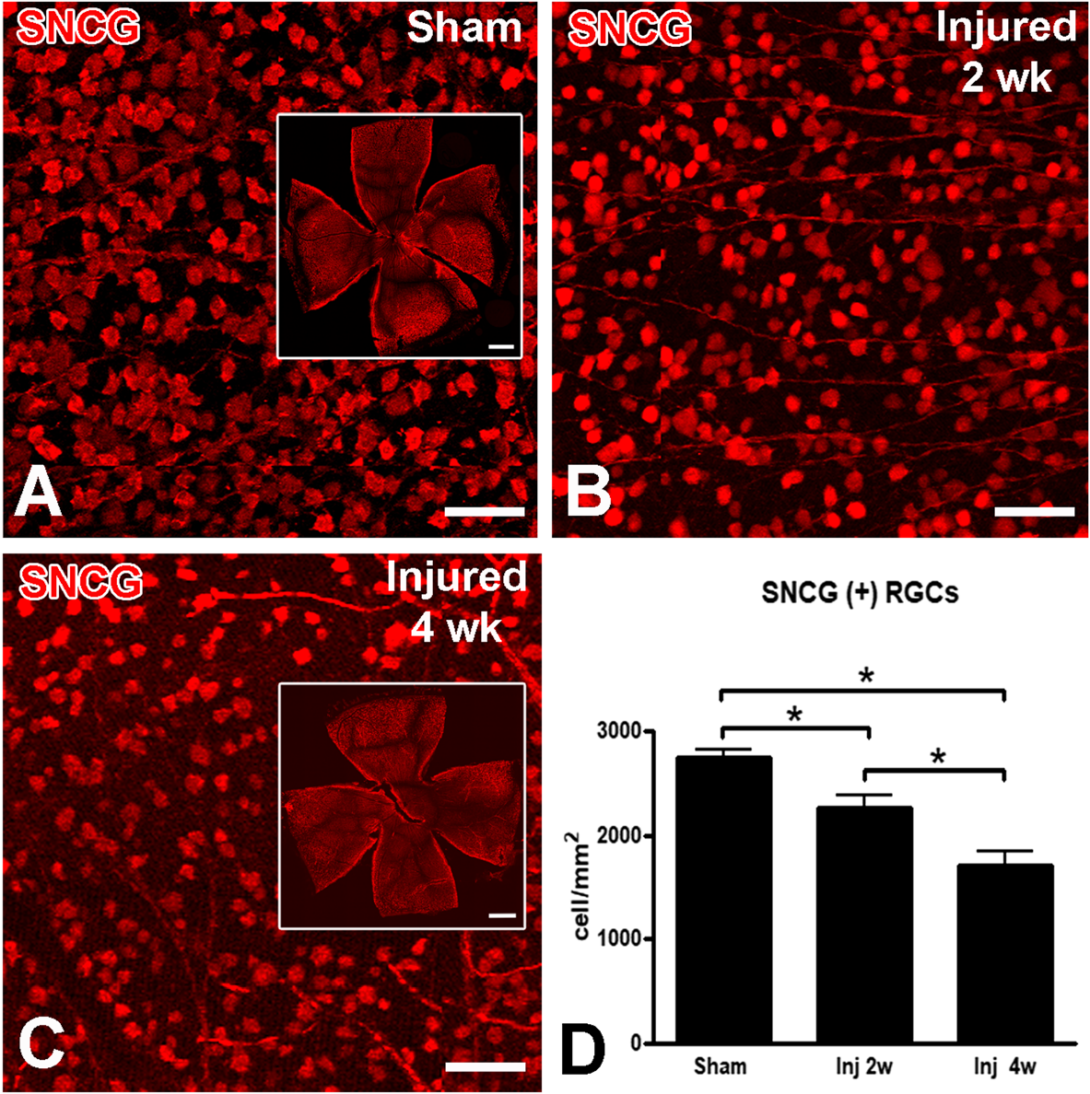
Progressive RGC loss after IA injury. In these whole-mount retinas stained with the RGC marker γ-synuclein (SNCG) 2 or 4 weeks after sham (**a**) or IA injury (**b**-**c**), there is evident RGC loss. Insets in **a** and **c** show the retinal flat mounts from which images in main panels originated. Note the progressive decrease in cell density from **a** to **c**. **d**. A bar graph with densities of surviving SNCG (+) RGCs 2 and 4 weeks post-injury, as compared to sham. Data were analyzed with one-way ANOVA followed by Tukey’s post hoc test. * *p* < 0.05. Scale bars: **a**-**c**, 50 μm; inserts in **a** and **c**, 100 μm

Seven days after injury, Gallyas silver staining revealed extensive axonal degeneration, from the optic nerve and tract to the superior colliculus (Fig. 2a-d). Degeneration was associated with the presence of deramified/hypertrophic microglia (Fig. 2e-f). Semithin sections of the optic nerve revealed numerous degenerative ovoids and axonal loss at 2 weeks post-injury that was maintained at 4 weeks (Figs. 3 and 4). Axonal degeneration profiles differ between the proximal part of the nerve, close to the eye, with the distal part of the nerve, close to the chiasm. The distal nerve has more pronounced dysmyelination and more severe axonal loss compared to proximal both at two- and four-weeks post injury. There is ongoing degeneration in the distal segment between two- and four-weeks post injury (Fig. 3d). At 4 weeks, de- and dys-myelination were less evident distally, but there was extensive myelin debris (Fig. 4c). To determine if IA caused RGC death, we counted the number of SNCG-positive cells in retinal flatmounts two- and four-weeks post-injury and we found a progressive decrease in the number of RGC somas in the course of the first month (Fig. 5). Taken together, these results suggest that the IA model produces a focal axonal injury that leads to progressive axonal and somal RGC degeneration.

### The DLK-JNK pathway is activated in RGCs in the course of visual TAI

**Fig. 6:**
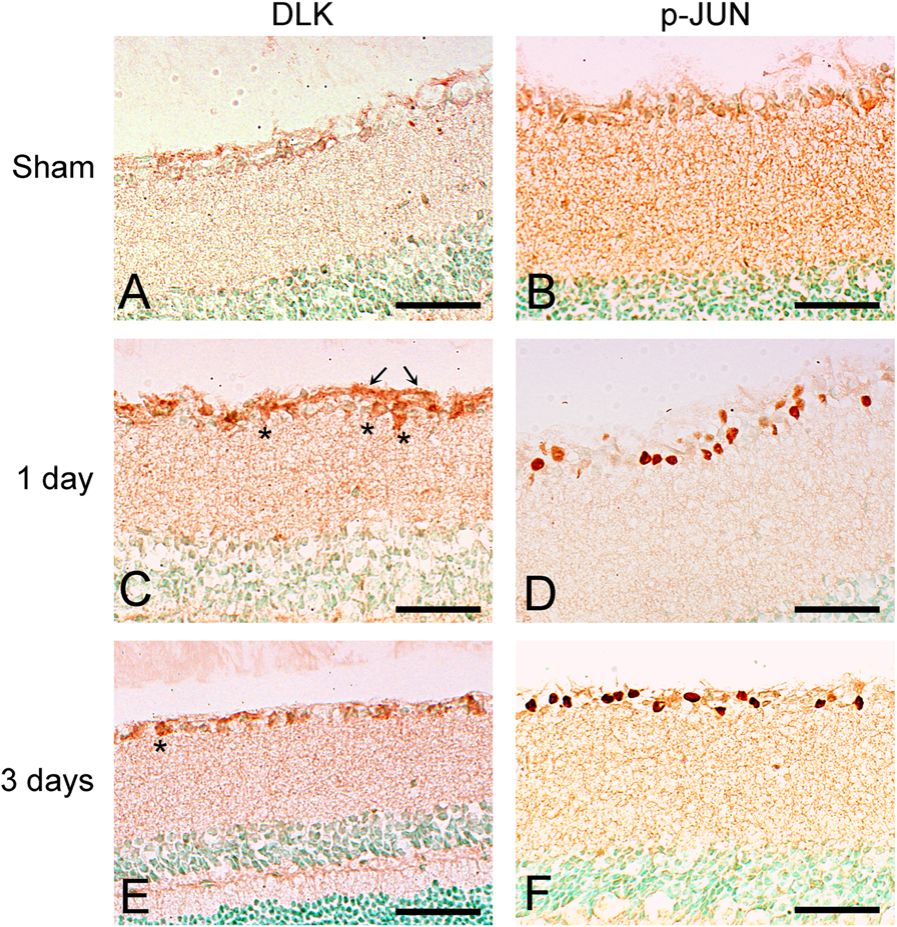
Activation of the DLK-JNK axis in the retinas of IA-injured mice. **a**, **c**, **e**. These horizontal retinal sections were processed for DLK immunohistochemistry and demonstrate the induction of DLK immunoreactivity in cell bodies (asterisks) in the ganglion cell layer and in axons in the nerve fiber layer (arrows) 1 and 3 days post-injury. There is very little immunoreactivity in sham retinas (**a**). **b**, **d**, **f**. These sections were immunostained for p-JUN and show the presence of many p-JUN (+) nuclei in the ganglion cell layer 1 and 3 days post-injury. Sham-injured retinas (**b**) are negative. Scale bars: **a**-**f**, 50 μm

**Fig. 7:**
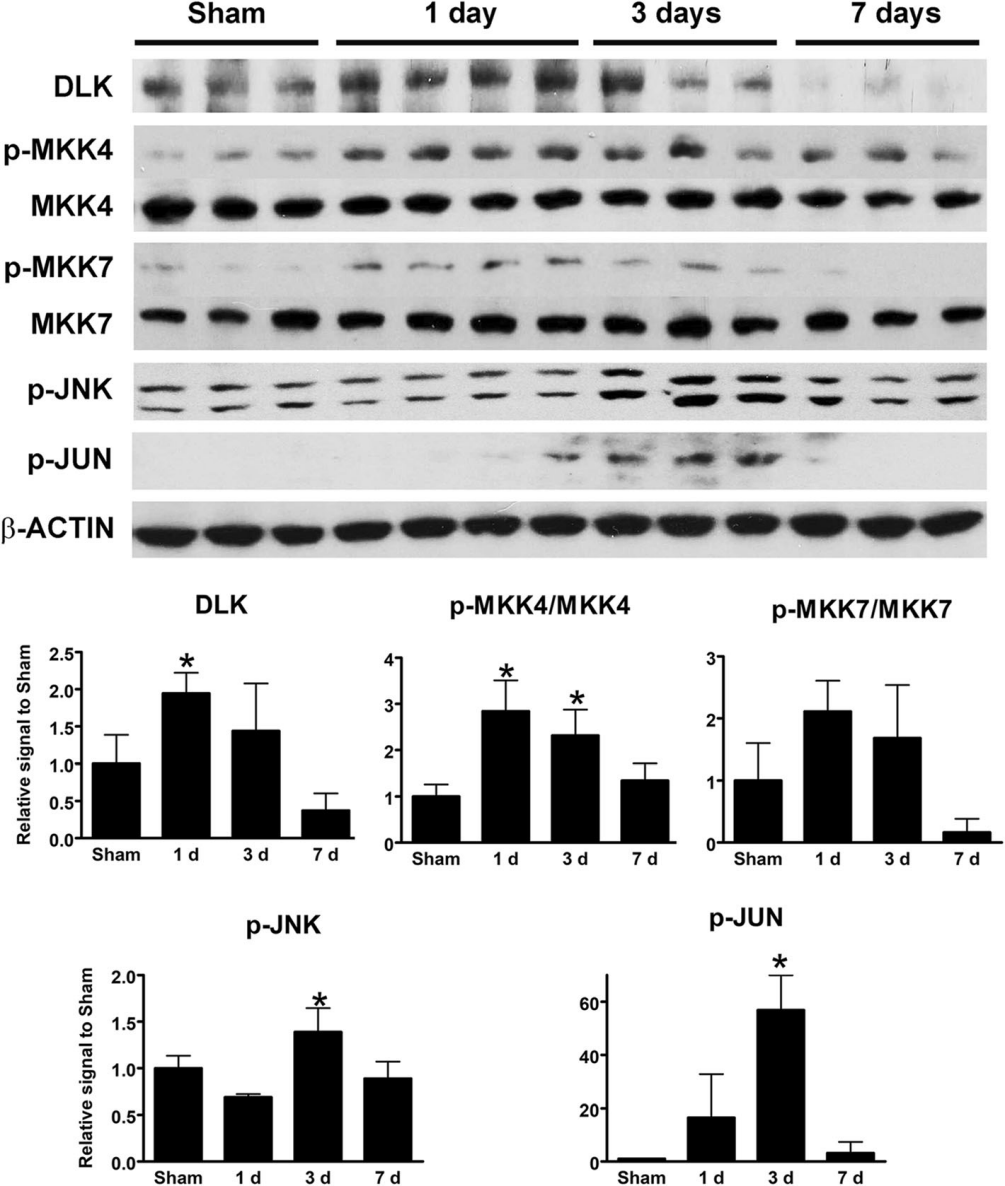
Expression of kinases in the DLK-JNK pathway in the retina after IA injury by Western blotting. Bar graphs at the bottom represent quantitation of the intensity of protein bands for DLK, activated MKK4 and MKK7 ratios, p-JNK and p-JUN in the sham condition and at 1, 3, and 7 days post-injury. Data were analyzed with one-way ANOVA followed by Tukey’s post hoc test. * *p* < 0.05

Based on previous work implicating DLK-JNK signaling in RGC degeneration after optic nerve crush, we tested whether a diffuse TBI model such as IA activates the DLK-JNK cascade in a similar fashion. Since DLK levels are indicative of pathway activation, we immunostained retinal sections for DLK and also immunoblotted whole retinal lysates. Both approaches showed transient upregulation of DLK, peaking around 1 day after injury (Fig. 6a, c, e and Fig. 7). We did not pursue LZK IHC or immunoblotting because of the lack of reliable antibodies. To determine if this upregulation was associated with pathway activation, we used immunoblotting and IHC to measure the phosphorylation state of the downstream kinases MKK4, MKK7, JNK1–3, and the transcription factor JUN. By 1 day and extending at least until day three after injury, increased phosphorylation of each of the DLK substrates, MKK4 and MKK7, was evident (Fig. 6b, d, f and Fig. 7). The time course of the activation was slightly different between IHC and immunoblotting, with whole-retina immunoblotting failing to detect the early increase likely because of the scarcity of RGCs in whole-retinal samples. Nonetheless, both techniques showed a robust activation of JUN after injury that returned to near baseline by 1 week (Fig. 7).

**Fig. 8:**
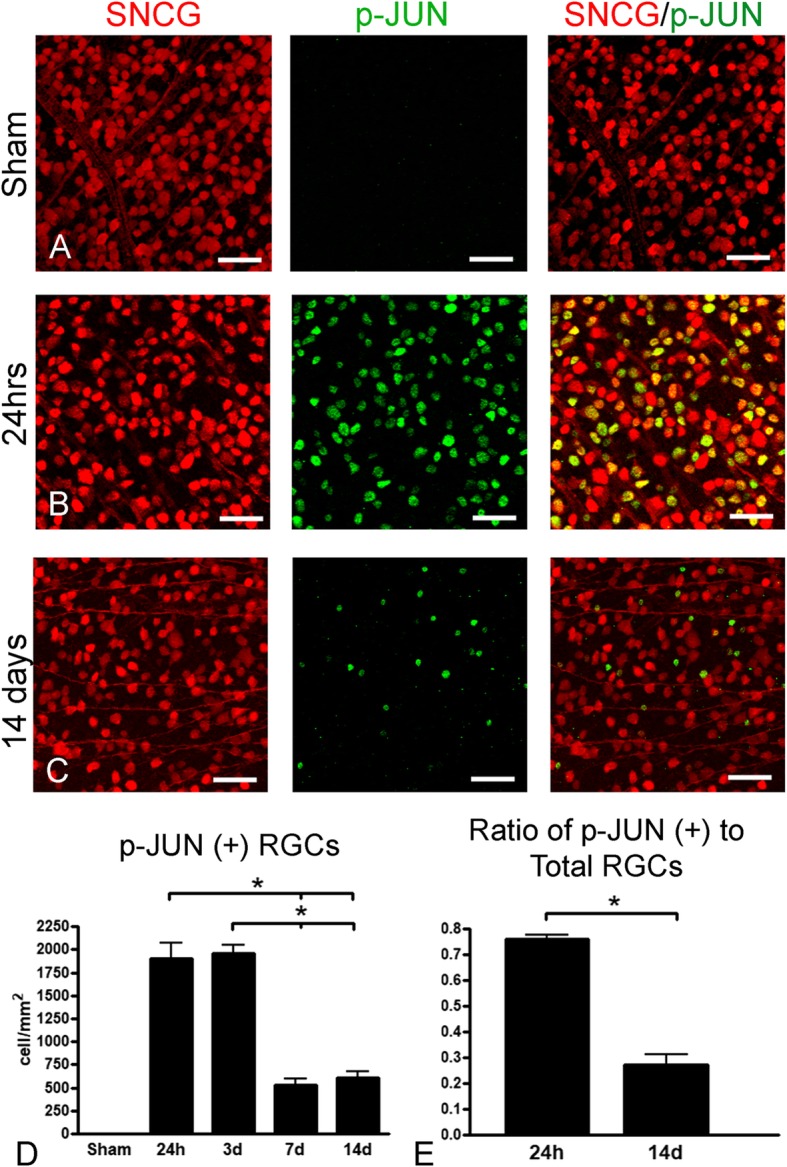
Quantitative assessment of DLK-JNK activation after IA injury based on counts of p-JUN (+) RGCs. **a**-**c**. In whole-mount retinas dually stained for the RGC marker γ-synuclein (SNCG) (red) and the DLK-JNK marker p-JUN (green), there is induction of p-JUN in RGCs at day 1 (**b**) and attenuation of labeling by day 14 (**c**). No p-JUN (+) RGCS are seen in the sham scenario (**a**). Left-sided images have been acquired with green filter combination for red SNCG immunofluorescence, images at the center have been acquired with blue filter combination for green p-JUN immunofluorescence, and panels on the right are merged images in which double-labeled profiles appear orange. Note the extensive colocalization at day 1, based on the large number of orange profiles (**b**, *right panel*). Most p-JUN (+) nuclei at day 14 seem to belong to smaller or atrophic RGCs (**c**). **d**-**e**. Bar graphs of densities of p-JUN (+) RGCs in the retinas of sham and injured animals at 1, 3, 7, and 14 days post-injury (**d**), and of the ratios of densities of p-JUN (+) RGCs over densities of total RGCs from two representative time points, i.e. 1 and 14 days (**e**). Significant differences are indicated with asterisks. The comparison in (**e**) was done to ensure that reduction in numbers of double labeled profiles in (**d**) was not simply an artifact of the progressive death of RGCs. Data were analyzed with one-way ANOVA followed by Tukey’s post hoc test for (**d**) and with student’s t-test for (**e**). * *p* < 0.05. Scale bars: 25 μm

One confounding possibility is that RGCs with active DLK signaling may die, thereby explaining the transient kinase activation. To test this hypothesis, we double stained retinal whole-mounts with antibodies against p-JUN for pathway activation and the RGC marker SNCG for viable neurons. The increased density of p-JUN-positive RGCs at one, three, seven, and 14 days after IA injury confirmed activation of the DLK-JNK axis (Fig. 8). Moreover, between one and 14 days, there was a decrease in the number of live RGCs with JUN activation, suggesting that kinase activation is transient for most RGCs. Because our TBI model initially activates DLK-JNK signaling in 75% of RGCs (Fig. 8d), but ultimately only ~ 40% of RGC somas and axons die (Fig. 5), it is likely that some fraction of RGCs are able to recover from the injury and normalize the DLK signal. Retinal ganglion cells with prolonged p-JUN expression appear atrophic and with decreased SNCG expression, an indication that they may be degenerating neurons. This potential recovery is surprising given that activation of the DLK-JNK pathway in the optic nerve crush model is sustained and leads to death of most RGCs [17]. In contrast, TBI leads to a robust initial activation of the DLK-JNK pathway, which is partially reversed in the post-acute phase of injury in surviving RGCs.

### The activation of JUN in visual TAI is dependent on DLK signaling

**Fig. 9:**
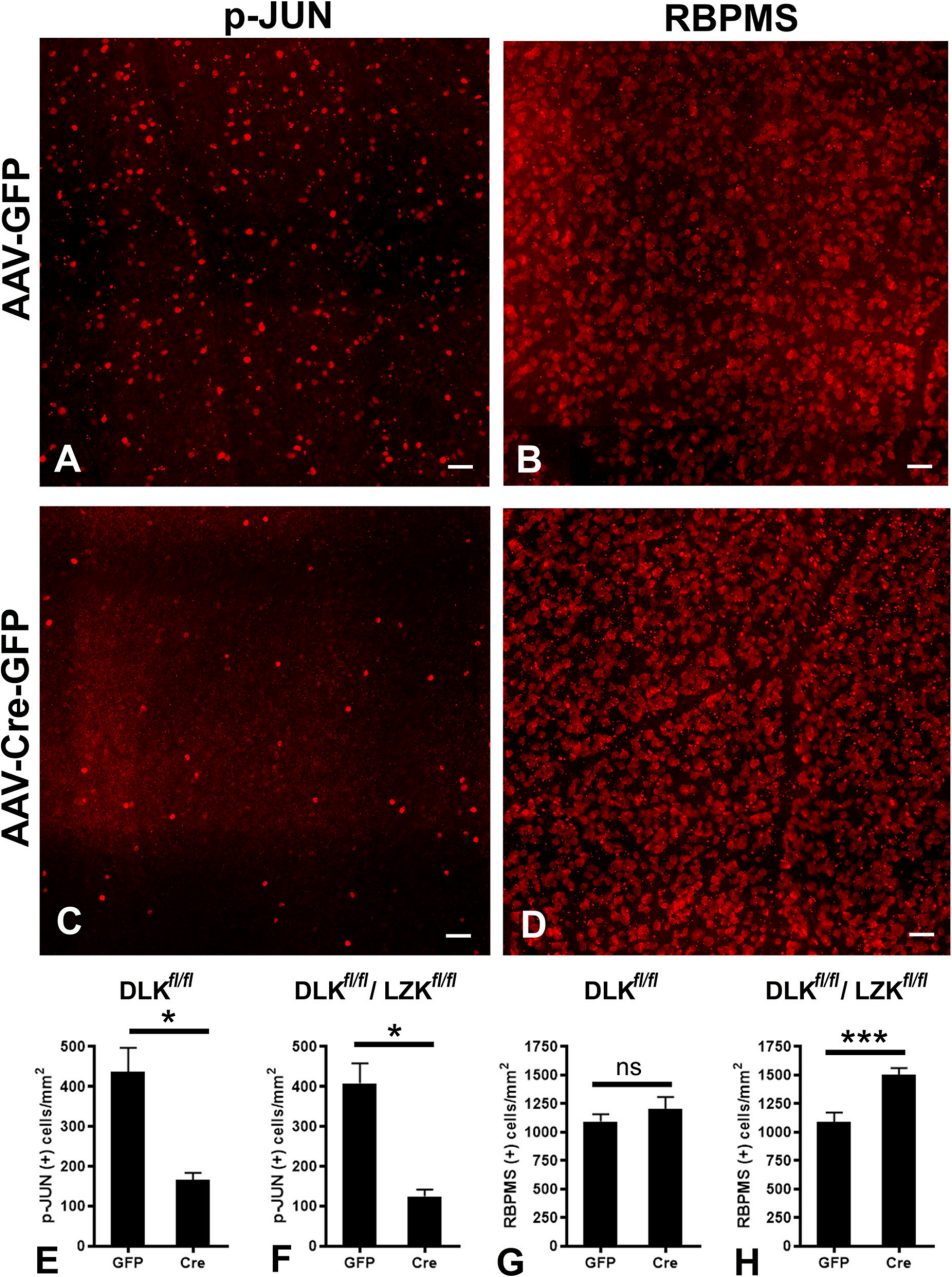
The effects of *Dlk* or combined *Dlk*/*Lzk* deletion on JNK signaling and RGC survival after IA injury. Subjects were *Dlk*^*fl/fl*^ or *Dlk*^*fl/fl*^*Lzk*^*fl/fl*^ mice. As elsewhere in this paper, p-JUN expression was used as a marker of DLK-JNK activation. Retinal ganglion cells were labeled with RBPMS. **a-d**. Representative images of p-JUN (**a**, **c**) and RBPMS (**b**, **d**) immunostained retinas in which *Dlk* and *Lzk* was deleted (AAV-Cre-GFP, **c**-**d**) and retinas from fellow eyes in which *Dlk* and *Lzk* were left intact (AAV-GFP, **a**-**b**). Images illustrate the suppression of p-JUN immunoreactivity at day 3 post-injury (**c**) and improved RGC survival 30 days post-injury (**h**) with combined *Dlk*/*Lzk* deletion. **e**-**h**. Bar graphs with quantitative assessments of the effects of *Dlk* or *Dlk*/*Lzk* deletion on p-JUN expression measured at day 3 after injury (**e** and **f**, respectively) and also the effects of *Dlk* or *Dlk*/*Lzk* deletion on RGC survival measured 30 days after injury (**g** and **h**, respectively). Scale bars: **a**-**d**, 25 μm

We have previously shown that DLK is the major MAP3K input responsible for JNK activation in response to axotomy in the optic nerve crush model, with additional contribution from LZK [18, 19]. To explore the degree to which the same kinases are responsible for JNK activation following the non-transecting injury in the IA model, we turned to mice with conditional alleles of *Dlk* and *Lzk* [18, 22]. Floxed *Dlk* or floxed *Dlk/Lzk* (*Dlk*^*fl/fl*^ and *Dlk*^*fl/fl*^*Lzk*^*fl/fl*^ mice, respectively) were intravitreally injected with AAV-Cre into one eye and a control virus (AAV-GFP) into the fellow eye. After 2 weeks, i.e. time sufficient for AAV to complete its lifecycle and trigger recombination, mice were subjected to TBI with IA. We then measured the rate of JUN phosphorylation as determined by the number of p-JUN (+) RGCs 3 days after injury (when signaling peaks based on immunohistochemical and immunoblot data). Compared to control animals (Fig. 9a), eyes with a targeted disruption of DLK/LZK had a significant (~ 75%) suppression of JUN phosphorylation (Fig. 9c, f). Interestingly, similar suppression was seen with DLK disruption alone (Fig. 9e), suggesting that DLK is a primary mediator of JNK activation in the IA model, and that LZK cannot compensate for a loss of DLK.

### Combined deletion of *Dlk* and *Lzk* increases survival of RGCs in visual TAI

To test the role of DLK and LZK in cell death following IA, a separate group of *Dlk*^*fl/fl*^ and *Dlk*^*fl/fl*^*Lzk*^*fl/fl*^ mice were injected with AAV-Cre or AAV-GFP and RGC survival was quantified 30 days after injury. Deletion of *Dlk* failed to show a significant effect on survival (Fig. 9g). However, combined deletion of *Dlk* and *Lzk* was protective and significantly increased survival of RGCs (Fig. 9b, d, h). These findings suggest that DLK and LZK have redundant abilities to trigger cell death in TAI, which differs from the ONC model in which *Dlk* deletion alone is robustly protective [18].

### Pharmacologic DLK/LZK inhibition improves RGC survival in visual TAI

**Fig. 10:**
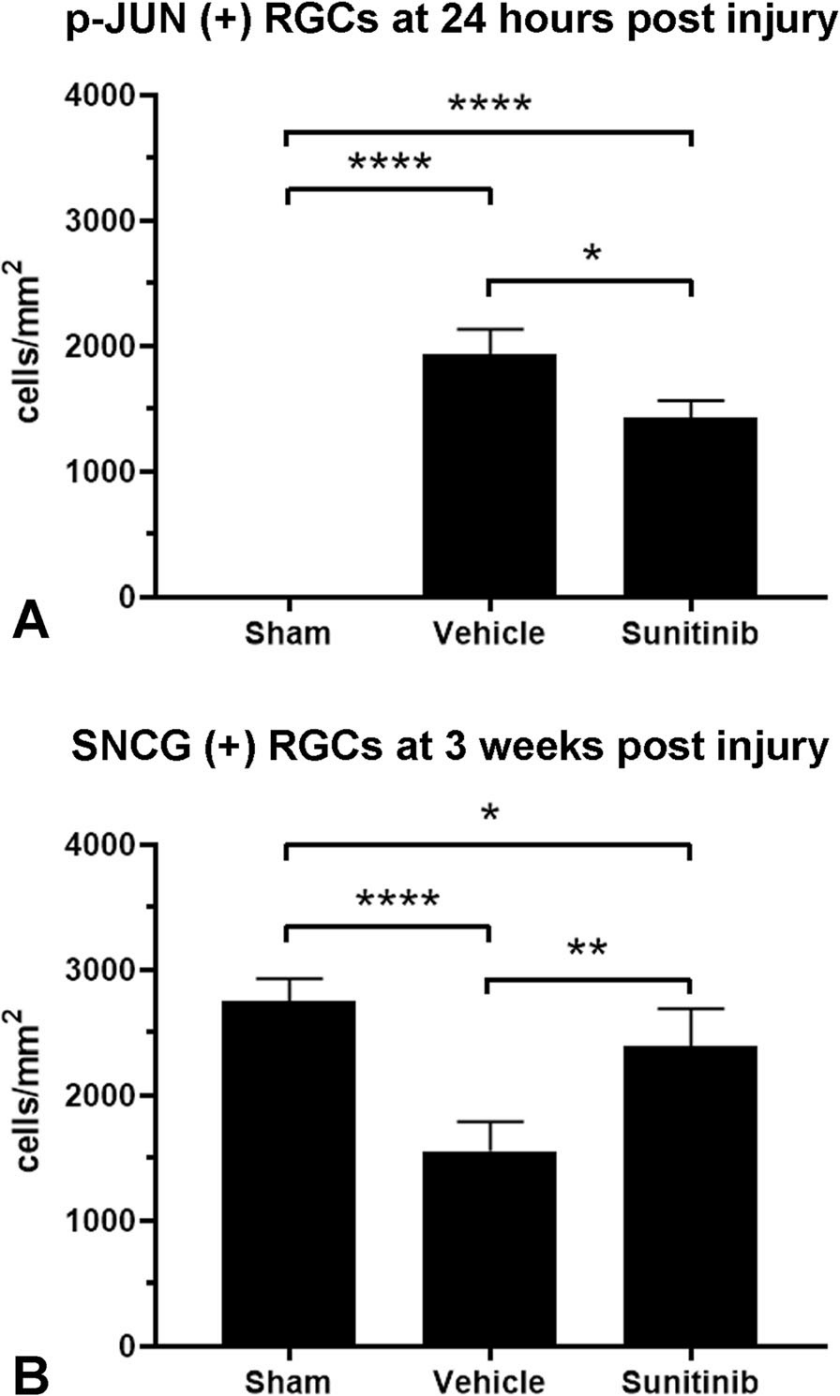
Suppression of JNK signaling and promotion of RGC survival by sunitinib in the IA model. **a** As elsewhere in this paper, DLK-JNK activation in retina was marked by density of p-JUN immunoreactivity in retinal flatmounts that here was measured at day 1 post injury. **b** Survival was based on the density of SNCG (+) cells that was measured 3 weeks post injury. Data were analyzed with one-way ANOVA followed by Tukey’s post hoc test. * *p* < 0.05, **p < 0.01, ***p < 0.001

Since genetic disruption of DLK/LZK protected injured RGCs, we asked whether pharmacological inhibition of DLK and LZK could similarly improve RGC survival in the IA model. Using published profiling data, we identified the FDA-approved protein kinase inhibitor sunitinib as having activity against DLK and LZK, but not against the downstream kinases MKK4, MKK7 and JNK1–3 [30]. Given that sunitinib penetrates the CNS [31], we tested whether its administration could promote RGC survival in the IA model. First, to confirm target engagement, mice were treated with intraperitoneal sunitinib (or the vehicle control) at 1 day and 4 h prior to injury, and then once more on the day after injury. Retinas were then harvested and stained for p-JUN. Sunitinib treatment led to a significant decrease in the number of p-JUN positive RGCs, confirming that it was able to partially suppress JNK signaling (Fig. 10). We then repeated the experiment, this time extending the treatment period for 21 days and assaying for RGC survival using SNCG staining of retinal whole-mount preparations. The results show that sunitinib treatment can improve neuronal survival following IA, increasing the number of surviving RGCs from 1500 cells/mm^2^ to nearly 2300 cells/mm^2^ (*p* < 0.05, *t*-test) when compared to vehicle, although the effect does not achieve normal (sham) RGC densities (Fig. 10b).

### Combined deletion of *Dlk* and *Lzk* protects optic nerve axons early in the course of TAI-associated axonopathy

**Fig. 11:**
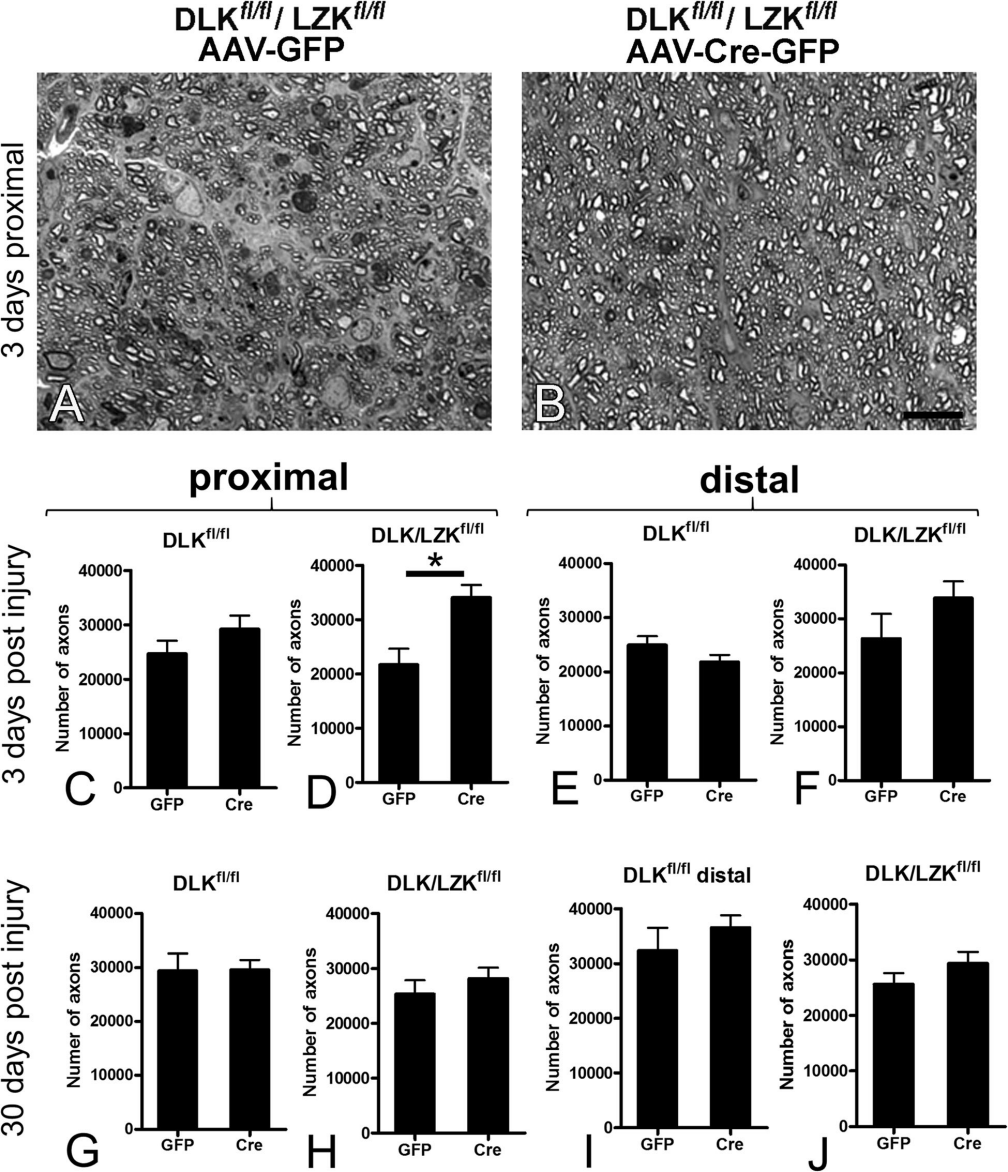
Combined *Dlk*/*Lzk* deletion using *Dlk*^*fl/fl*^*Lzk*^*fl/fl*^ mice delays axonal degeneration in the optic nerve. **a**-**b**. Representative images from semithin sections of the proximal optic nerve 3 days post injury showing the protective effect of *Dlk*/*Lzk* deletion. Cre-treated optic nerve is depicted on the right (**b**) and control optic nerve from the fellow eye is shown on the left (**a**). **c**-**j**. Bar graphs with counts of axons in the optic nerve proximal (**c**-**d** and **g**-**h**) and distal (**e**-**f** and **i**-**j**) to the site of the initial traumatic disruption three (**c**-**f**) and 30 (**g**-**j**) days post injury in *Dlk*^*fl/fl*^ (**c**, **e**, **g**, **i**) or *Dlk*^*fl/fl*^*Lzk*^*fl/fl*^ (**d**, **f**, **h**, **j**) mice treated with Cre or control (GFP) vectors in the two eyes. There are eight combinations of genotype × proximal or distal location × early or late time point post injury. Of all combinations, the only significant effect on axonal degeneration is that of the deletion of both *Dlk* and *Lzk* in the proximal nerve early post injury (**d**). Data were analyzed with student’s t-test. * *p* < 0.05. Scale bars: **a**-**b**, 25 μm

Having established the long-term protective effect of either genetic or pharmacological DLK/LZK inhibition on RGC survival, we explored the role of these kinases in axonal degeneration produced by the IA model. Based on stereological counts of myelinated axons in the optic nerve proximal and distal to the putative site of injury and comparisons between AAV-Cre and control AAV-GFP-treated eyes, DLK deletion alone did not appear to protect axons at 3 or 30 days post-injury. The combined deletion of *Dlk* and *Lzk* protected optic nerve axons at 3 days after TBI, but this effect was not sustained at the 30-day time point post injury (Fig. 11). Taken together, these results suggest that TAI-associated axonal degeneration in the visual system is not as dependent on the DLK/LZK genetic program.

## Discussion

Our study explores the role of two MAP3Ks, DLK and LZK, in TAI-associated axonopathy and RGC degeneration in the visual system following IA injury. Traumatic axonal injury in the visual system is featured by axonal transport defects followed by axonal swellings and bulbs in a segment of optic nerve. This is then followed by progressive axonopathic changes (traumatic axonopathy) leading to degeneration of a sizable portion of axons and retrograde death of RGCs. Our findings indicate that TAI in the visual system is associated with the activation of the DLK/LZK-JNK axis that is responsible, at least in part, for RGC somal death, although its role in axonal degeneration is limited. The conditional knockout of *Dlk* and *Lzk* in adult mice employed here precludes a neurodevelopmental compensation effect on RGC survival and points to the central role of the DLK-JNK axis in neurodegeneration after TBI. The role of DLK/LZK in triggering RGC degeneration during TAI is further supported by the neuroprotective effect of the FDA-approved kinase inhibitor, sunitinib, a finding with therapeutic potential. Our results show that molecular signals operating in simple forms of axonal injury, i.e. axotomy, play important roles in some of the degenerative outcomes of TAI in the CNS.

We have previously shown that our diffuse model of TBI (IA) leads to multifocal TAI in the corticospinal tract, optic nerve, gracile fasciculus, fornix, and corpus callosum [11, 12]. Traumatic axonal injury in the visual system is associated with optic nerve axonopathy (traumatic axonopathy) that is further characterized here and has been shown to correlate with injury burden [11]. The primary biomechanical disruption of the optic nerve corresponds to a region between the orbital apex and the chiasm which is roughly used for the pathological distinctions between “proximal” and “distal” in this paper. Degenerating optic nerve axons show widespread dysmyelination/demyelination that is evident as early as 3 days post-injury. Axonopathy is more severe distal to the site of biomechanical disruption where it continues to evolve over the course of the 4-week period examined in our study. Compared to the effects of IA in other CNS tracts, e.g. the corticospinal tract, a distinct feature of TAI in the optic nerve is the retrograde degeneration of neurons (RGCs), in keeping with the classical vulnerability of these nerve cells to death after axonal lesions [32–34]. This notion is further supported by the fact that progression of death of RGC somata appears to lag behind the wave of axonal degeneration. The co-registration of rates of death in RGCs and in proximal axons, both of which are ~ 25–30%, may suggest a biological correlation between degeneration of perikarya and degeneration of proximal axons.

DLK is part of a highly-conserved retrograde injury signal that is triggered by axotomy, leading to the activation of both apoptotic and regenerative processes [17, 19, 21, 35]. However, simple axotomy models (e.g. optic nerve crush) differ from more clinically relevant models, e.g. TAI, in that the latter tend to produce more graded and incomplete axonal injury. The degree to which DLK is required for TBI- and TAI-associated axonopathy had not been previously explored. Our results suggest some key similarities and differences. As with axotomy, TAI leads to upregulation of DLK and activation of the MKK/JNK/JUN signaling pathway in RGCs. Moreover, DLK acts as obligatory upstream mediator of JUN activation, because targeted disruption of DLK leads to a near-total suppression of JUN phosphorylation. Perhaps the most important difference is that the TAI model triggers DLK activation in such a way that a subset of RGCs can recover and terminate the signal. The mechanism by which this phenomenon occurs is probably related to subthreshold biomechanical injury in a large number of optic nerve axons but may also involve selective vulnerability of subclasses of RGC neurons. These issues need to be explored further because they may have important implications for RGC neuroprotection in the context of TBI.

Furthermore, in contrast to axotomy models in which deletion of *Dlk* by itself rescues the survival of ~ 75% of RGCs [16, 17, 19] in the TAI model, knockout of *Dlk* has little effect on RGC survival. Only when both DLK and LZK were targeted did we see an increase in RGC survival, suggesting a degree of genetic redundancy for these two highly-related kinases. Not surprisingly, the degree of redundancy seems to be dependent on the model, with the TAI model and primary RGC cultures showing a large degree of DLK/LZK redundancy (i.e. combined inhibition is required for robust survival), while the ONC model shows a lesser degree of redundancy (i.e. combined disruption produces a more modest increase in survival over *Dlk* disruption alone). It is also interesting to note that deletion of *Dlk* with or without *Lzk* leads to a significant reduction in the downstream phosphorylation of JUN but only the combined deletion of the two protects RGC viability. Although we cannot exclude effects on the kinetics or localization of JNK activation, these data could indicate that LZK may be upstream of an as-yet-unidentified pathway responsible for RGC cell death in TAI.

In order to develop an expedited route to clinically evaluate DLK/LZK inhibition as a neuroprotective strategy, we previously surveyed FDA-approved protein kinase inhibitors for ones with activity against DLK and LZK. This approach identified sunitinib (Sutent®), FDA-approved for renal cell carcinoma and gastrointestinal stromal tumor, as having high nanomolar IC_50_ for both DLK and LZK. Moreover, we showed that sunitinib increased the survival of induced pluripotent stem cell (iPSC)-derived human RGCs [18]. Here we have extended these findings to demonstrate that pharmacological blockade of DLK and LZK with sunitinib increases RGC survival in vivo in a rodent TBI model. To these authors’ knowledge, and with the possible exception of antioxidants [36], this is the first demonstration of an FDA-approved compound decreasing neuronal cell death in a TBI model.

Central nervous system tracts other than the visual system that are vulnerable to TAI and succumb to traumatic axonopathy [12] may also use DLK-dependent injury signals; therefore, our findings raise the prospect of using protein kinase inhibitors as part of a neuroprotective strategy for TBI. Traumatic brain injury very commonly affects axons and the state of axons after injury is important for the integrity of neuronal circuitry and the function of neural networks [37]. After we established that RGC somatic death is, at least in part, a DLK/LZK-dependent phenomenon, we explored the effect of the DLK/LZK pathway on axonal survival. DLK appears to be implicated in axonal degeneration through a negative effect on levels of SCG10, an anterogradely transported protein that promotes axonal survival after axotomy in vitro [38]. More recently, the involvement of DLK in axonal degeneration has been examined in vitro using primary dorsal root ganglion neurons [23]. These experiments have revealed an important role of DLK in maintaining basal levels of NMNAT2, an essential axonal survival factor, through the degradation of the palmitoylated form of the enzyme under normal conditions. Deletion of *Dlk* raises basal level of anterogradely transported NMNAT2 prior to injury, and thus delays axonal degeneration. This protective effect is more pronounced when combined with deletion of the *Phr1/Skp1a/Fbxo45* ligase complex that is important for the degradation of the nonpalmitoylated fraction of NMNAT2 [23]. Consistent with these observations, we found that the combined deletion of *Dlk* and *Lzk* only protected optic nerve axons from degeneration early, i.e. 3 days, post injury. To our knowledge, this is the first demonstration that LZK plays a role, albeit limited, in an active axonal degeneration program. This finding is in line with the kinetics of NMNAT2, a labile protein that needs continuous replenishment to exert protective effects on axons [39]. The blockade of anterograde transport that occurs with TAI would have prevented the axonal transport of NMNAT2, leaving the axon only with basal levels of the protein, which may be higher is neurons with deleted *Dlk/Lzk*. This effect, however, may not be sufficient to completely protect injured axons, as suggested by the fact that there is no significant effect on survival at 30 days post injury.

## Conclusion

Here we have shown the RGC loss after TBI can be prevented, at least in part, by the genetic deletion or pharmacological inhibition of DLK/LZK. This finding may be an important step in protecting RGCs in the context of TBI and potentially allowing them to reintegrate into the visual circuitry if many axons are still relatively intact or if regenerative strategies become more effective in the future [40]. We also showed that by blocking DLK/LZK we may delay degeneration of RGC axons although, as has been also shown in simple axotomy lesions, the effect on RGC somata is clearly more robust [16, 41–43]. Finally, here we showed that an FDA-approved drug, sunitinib, with activity against both DLK and LZK, is able to increase RGC survival in a TBI model. Although future work will need to establish if pharmacologic DLK/LZK inhibition can prevent degeneration when given in a more clinically-relevant post-injury paradigm, our findings already suggest that this intervention may be a promising lead for translational efforts in the field of TBI.

## Data Availability

The primary data used and/or analysed during the current study (e.g. images of the original immunoblots) are available from the corresponding author on reasonable request.

## Abbreviations

APP: Amyloid precursor protein
BBB: Blood brain barrier
CTB: Cholera toxin B
DAB: 3,3′-diaminobenzidine
DAPI: 4′, 6-diamidino-2-phenylindole
DLK: Dual leucine zipper kinase (MAP3K12)
IA: Impact acceleration
IHC: Immunohistochemistry
JNK: JUN N-terminal kinase
LZK: Leucine zipper kinase (MAP3K13)
MAP2K: Mitogen activated protein kinase kinase
MAP3K: Mitogen activated protein kinase kinase kinase
MAPK: Mitogen activated protein kinase
MKK4: Mitogen activated protein kinase kinase four
MKK7: Mitogen activated protein kinase kinase seven
NMNAT2: Nicotinamide mononucleotide adenylyl transferase two
ON: Optic nerve
ONC: Optic nerve crush
OT: Optic tract
p-JUN: phosphorylated JUN
RBPMS: RNA binding protein, mRNA processing factor
RGC: Retinal ganglion cell
SC: Superior colliculus
siRNA: small interfering RNA
SNCG: Gamma synuclein
TAI: Traumatic axonal injury
TBI: Traumatic brain injury
